# Platforms for Optogenetic Stimulation and Feedback Control

**DOI:** 10.3389/fbioe.2022.918917

**Published:** 2022-06-08

**Authors:** Sant Kumar, Mustafa Khammash

**Affiliations:** Department of Biosystems Science and Engineering (D-BSSE), ETH Zürich, Basel, Switzerland

**Keywords:** optogenetics, optogenetic platform, light stimulation device, light-control, feedback control, *in silico* control, cybergenetics

## Abstract

Harnessing the potential of optogenetics in biology requires methodologies from different disciplines ranging from biology, to mechatronics engineering, to control engineering. Light stimulation of a synthetic optogenetic construct in a given biological species can only be achieved *via* a suitable light stimulation platform. Emerging optogenetic applications entail a consistent, reproducible, and regulated delivery of light adapted to the application requirement. In this review, we explore the evolution of light-induction hardware-software platforms from simple illumination set-ups to sophisticated microscopy, microtiter plate and bioreactor designs, and discuss their respective advantages and disadvantages. Here, we examine design approaches followed in performing optogenetic experiments spanning different cell types and culture volumes, with induction capabilities ranging from single cell stimulation to entire cell culture illumination. The development of automated measurement and stimulation schemes on these platforms has enabled researchers to implement various *in silico* feedback control strategies to achieve computer-controlled living systems—a theme we briefly discuss in the last part of this review.

## 1 Introduction

**FIGURE 1:**
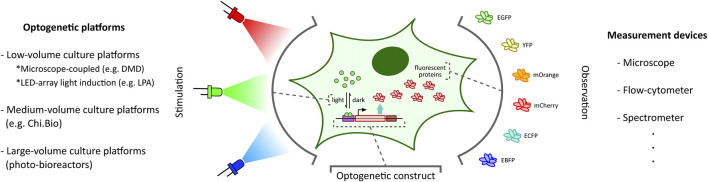
Optogenetic stimulation and target output observation in cellular context. Optogenetic studies involve placing a desired genetic construct under a light-activated tool (Kolar et al., 2018) (e.g. light-inducible transcription factors (Benzinger and Khammash, 2018) illustrated here) with an observable target output (e.g. fluorescent proteins (Rodriguez et al., 2017) illustrated here). Depending on the application requirements, one can choose from a plethora of optogenetic platforms (left: DMD-based platform (Rullan et al., 2018), LPA (Gerhardt et al., 2016), Chi.Bio (Steel et al., 2020)) and measurement devices (right) to stimulate and investigate the optogenetic constructs, respectively.

Recent progress in synthetic biology has established light as a leading tool for observation as well as stimulation of synthetic constructs in cellular contexts (MacDonald and Deans, 2016; Toettcher et al., 2011a; Deisseroth, 2011; Müller et al., 2015; Krueger et al., 2019; Baumschlager and Khammash, 2021; Gheorghiu et al., 2021; Forlani and Di Ventura, 2021; Pérez et al., 2021). Light can be precisely localized in space and instantaneously turned on and off. Achieving this degree of spatio-temporal control establishes light as an exceptionally attractive alternative to other existing stimulation techniques such as chemical induction (Fracassi et al., 2016). Inspired by photosensitive proteins found in nature, synthetic biologists have engineered phototosensors and photoreceptors into living cells to control cellular processes such as gene expression (Müller et al., 2015) (Figure 1) and protein-protein interaction (Toettcher et al., 2011b). This use of light to influence cellular processes is commonly referred to as optogenetics (Toettcher et al., 2011a; Deisseroth, 2011). Several optogenetic tools, pertaining to different cell types and spanning different wavelengths of light, have been developed and studied in the last decade (Tischer and Weiner, 2014; Müller et al., 2015; Repina et al., 2017; Kolar et al., 2018; Baumschlager and Khammash, 2021; Gheorghiu et al., 2021; Forlani and Di Ventura, 2021; Pérez et al., 2021). Applications of these optogenetic tools have been expanding rapidly in various disciplines, such as developmental biology (Johnson and Toettcher, 2018; Krueger et al., 2019).

The ability to provide accurate, tunable, and controlled delivery of light is crucial for the effective application of optogenetics. In recent years, there has been a rapid acceleration in the development of new strategies and hardware-software platforms fulfilling this requirement (Figure 1). Custom-designed light stimulation devices (*optogenetic platforms*) targeted at different cell culture volumes are now available to address the specific needs of various applications. Certain specialized devices can further facilitate light induction capability at intracellular resolution and support high-throughput operation. Many of these platforms also include dedicated devices to measure cellular outputs such as fluorescent protein reporters (Rodriguez et al., 2017) (Figure 1) or cell growth, enabling real-time observation of target cell behaviour in response to light stimulation.

**FIGURE 2:**
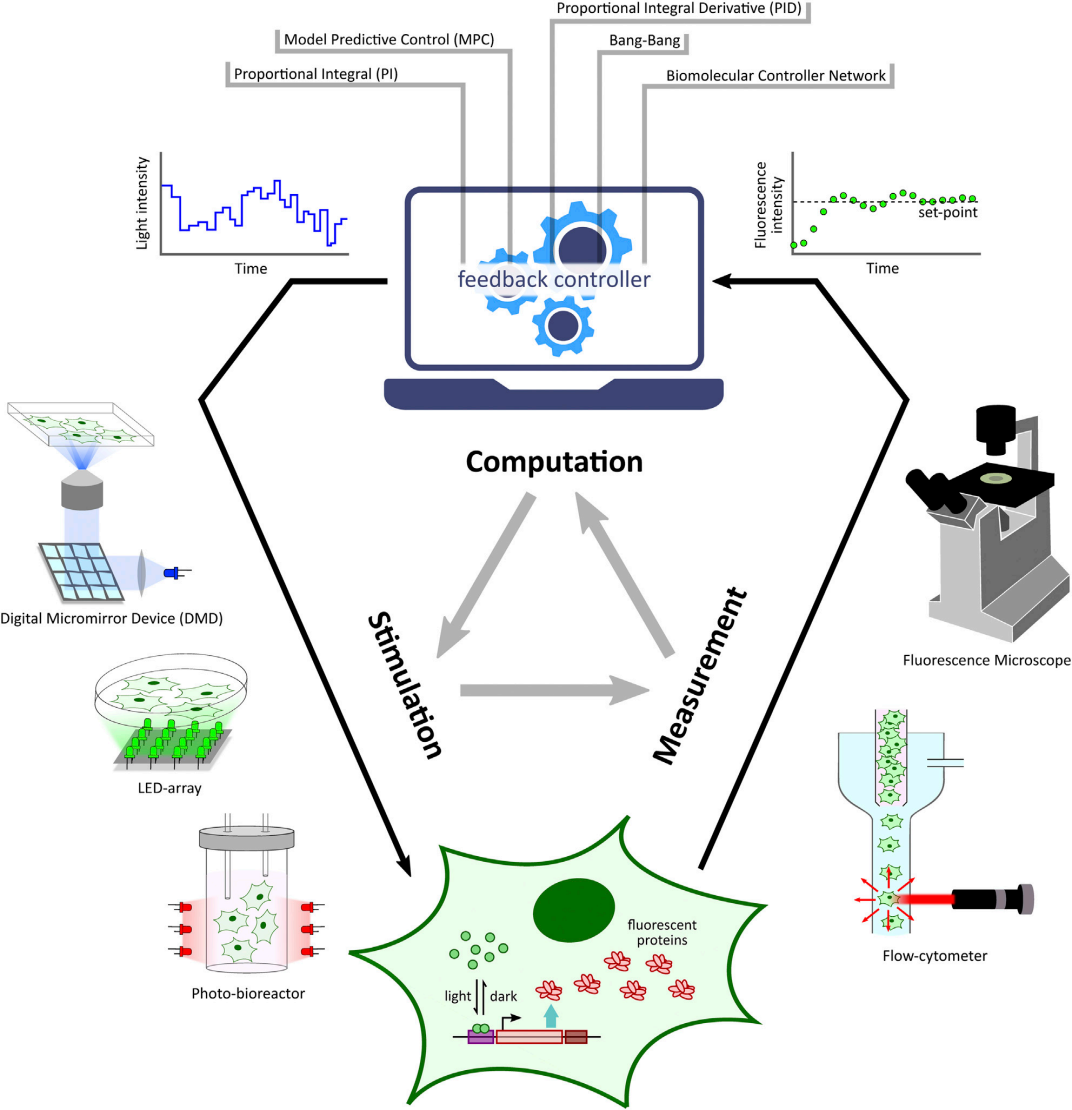
Optogenetic *in silico* feedback control framework. Given a target cell (engineered with desired optogenetic constructs and fluorescent reporter) culture, fluorescence measurements from target cells (measured *via* specialized devices, e.g. microscope, flow-cytometer, *etc*.) are sent to a computer where they are quantified. The quantified output is then used to run a controller (e.g. PI, MPC, *etc*.) simulation. The controller computes the required light input intensity for cells in order to achieve a desired output behaviour, such as tracking a desired set-point. This input light intensity is then applied to the target cells *via* a suitable light stimulation platform, thus closing the feedback loop. This measurement-computation-stimulation loop is performed at fixed intervals driving the output dynamics as desired.

Optogenetic platforms equipped with a combination of light stimulation and cellular activity measurement devices have allowed for the implementation of *in silico* feedback control (Figure 2) of cellular processes ranging in application from simple gene expression regulation (Kumar et al., 2021) to multicellular morphogenesis studies (Hartmann et al., 2020). An *in silico* feedback control loop first involves the quantitative measurement of a desired cellular output (e.g. fluorescence) by a suitable measurement device. The output measurement data are then sent to a control computer which performs dedicated computations over the data using *feedback controllers* (Figure 2) such as integral and model predictive control strategies to determine a light intensity that the target cells should be stimulated with in order to achieve a desired output behaviour (e.g. set-point tracking). The computed light intensity is then applied to the target cells *via* the light stimulation device. This sequence of measurement, computation, and stimulation are executed iteratively at fixed time intervals to achieve closed-loop feedback control of the target process.

This review aims to introduce and describe various optogenetic platforms and *in silico* control strategies developed for synthetic biology studies. In this article, we first categorize these platforms into three broad groups based on their intended target cell culture volume ( <1ml – low; 1–100 *ml*–medium; >100ml – large), and discuss their design methodologies in their respective applications, before offering our perspective on their further development. We further review a few unique platform designs intended for emerging optogenetic applications such as in animal implants and 3-D spatial induction. Finally, we conclude with a brief discussion on existing *in silico* feedback control strategies and applications. For brevity, the main focus is on optogenetic platforms demonstrated for non-neuronal cell studies, and platforms dedicated for neuronal (Warden et al., 2014; Kim et al., 2017; Vázquez-Guardado et al., 2020) and plant (Ochoa-Fernandez et al., 2020) cell studies are not discussed.

## 2 Low-Volume Culture Platforms

This category spans optogenetic platforms with target cell culture volumes ranging from a few microliters to a milliliter. These platforms are usually employed for short-term experiments to characterize and optimize optogenetic tools and downstream genetic circuits. They are also effective in studies such as pattern formation, requiring light-stimulation and observation at intra-/inter-cellular resolution. These platforms can be further divided into two groups: set-ups having a light stimulation device coupled with microscope systems, and set-ups having a dedicated LED-array light induction device adapted for micro-multiwell plates. The first group of platforms mostly facilitates higher spatial resolution and dynamic studies, whereas the second group is usually considered in high-throughput experiments pertaining to steady-state studies.

### 2.1 Microscope-Coupled Optogenetic Platforms

Light microscopes, by design, have suitable episcopic or diascopic light sources for transmitted-light, reflected-light, or fluorescence imaging. Without requiring any additional hardware modification, these imaging light sources can also be directly employed for optogenetic stimulation within the microscope field of view. In (Kennedy et al., 2010), the authors used fluorescence imaging blue (488-nm) light source, installed on their microscope, for stimulation purpose, and demonstrated light-induced activation of transcription and DNA recombination. A similar idea was adopted by Toettcher et al., 2013, where the authors used a 650-nm LED light source placed at the episcopic illumination port of the microscope for field of view stimulation, and ensured global 750-nm light illumination *via* the discopic side. This configuration allowed them to control both the activation (with 650-nm) and inactivation (with 750-nm) of PhyB (phytochrome B)-PIF (phytochrome-interacting transcription factor) interaction under a microscope. Further, in (Araki et al., 2014), the authors devised an automated fluorescence microscopy system in which they used fluorescence excitation light source for photoactivation and used a motorized field diaphragm in the fluorescence pathway to reduce the target area of illumination in the microscope field of view. Although having a simple design with precise control over illumination timing or duration, these direct episcopic/diascopic illumination microscope-coupled platforms have extremely limited utility for spatially-localized delivery of light. One way to achieve precise spatial control over light stimulation is by integrating sophisticated projection hardware with the microscope.

**FIGURE 3:**
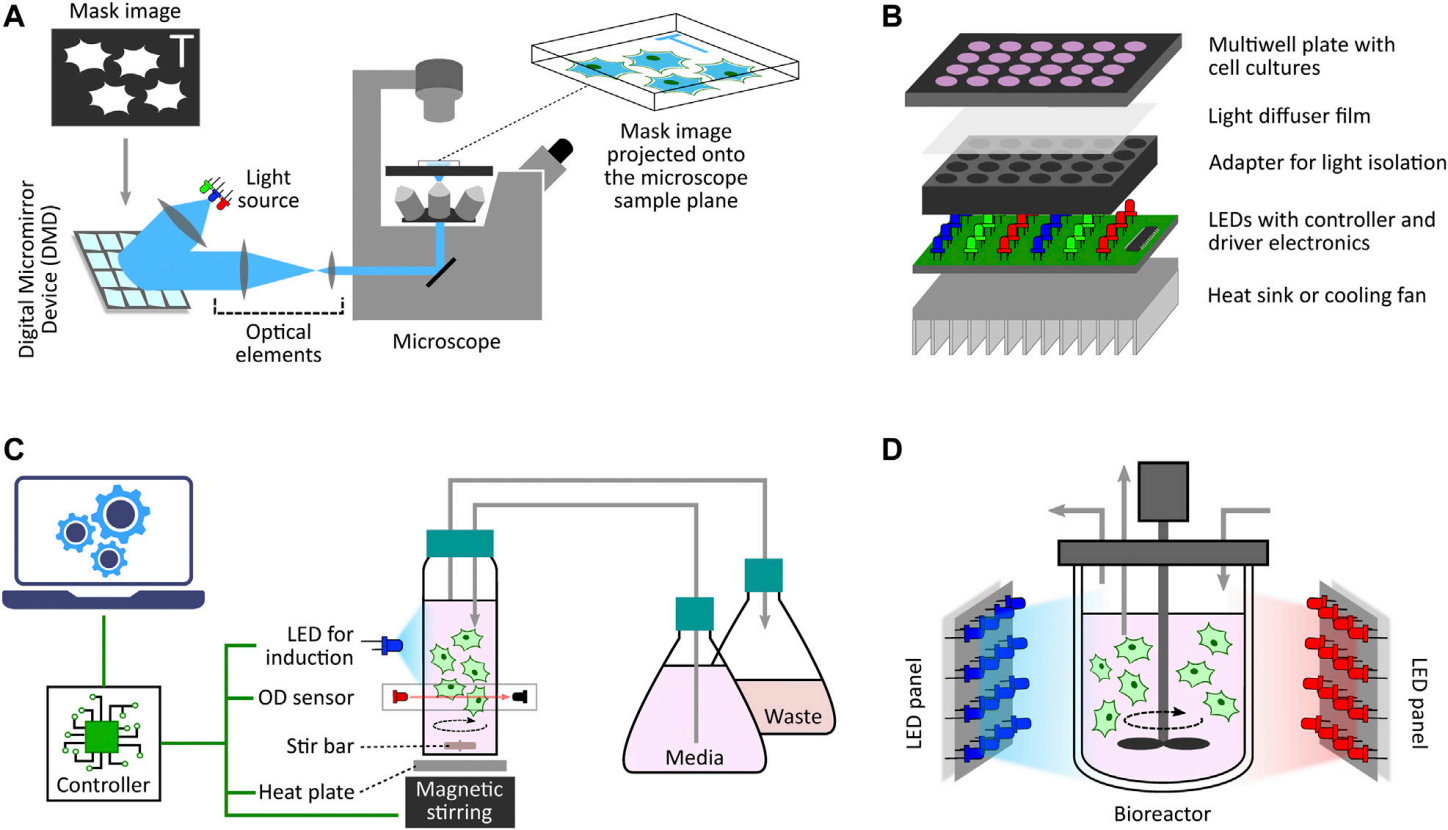
Diverse optogenetic platform architectures. **(A)** Microscope-coupled DMD-based platform illustration. The illumination light from LEDs is steered to fall onto a DMD at a particular angle. A DMD has micrometer-sized mirrors that can be individually tilted to ON and OFF positions. ON position allows the incident light to follow a defined path towards the microscope opening port whereas OFF position lets the incident light divert away after reflection. Once a given mask image is sent to the DMD controller, it sets the tilt-position of all DMD micromirrors accordingly with each micromirror representing an individual pixel of the image. These micromirrors oscillate between ON and OFF positions at a very high frequency, and the duty cycle of each micromirror is determined by the corresponding pixel intensity (gray value) in the mask image. When incident light is applied, the oscillating micromirrors allow the projection of the mask image towards the microscope opening port. The projection light then follows a series of optical elements (lens, filters, apertures, *etc*.) before entering the microscope port which then guides it towards the microscope objective lens. All optical elements are chosen and positioned in such a way that the projection image is focused onto the microscope sample plane allowing spatio-temporal and graded illumination of target cells placed under the microscope field of view. **(B)** Microtiter multiwell plate LED array illustration. These devices, in general, have a layered structure. The main layer is the electronic board fit with LEDs at regular matrix locations aligned with the target plate wells. This board can have multiple LEDs grouped together to provide multiple wavelengths per well. It also contains LED driver electronics and micro-controllers needed to set LED intensities and turn them ON/OFF as and when desired in a pre-programmed manner. To provide independent illumination to each well, an opaque adapter can be placed on top of the LED board isolating individual LEDs. The multiwell plate containing target cell culture is then placed on top of the adapter such that each well receives light from the LED underneath independent and isolated from other wells. One can also place a light diffuser film between the plate and the adapter to provide more homogeneous illumination within each well. Furthermore, if required, one can also add a heat sink or other cooling system under the LED board to dissipate heat generated by the electronics board which may adversely effect the target cell culture. **(C)** Milli-culture optogenetic platform illustration. The target cell culture is usually placed in a glass vial which can be externally illuminated with LEDs. Cell culture incubation temperature is maintained with a heat plate below the vial or a heated water bath. Aeration is achieved using devices such as magnetic stirrers. Some platforms also have an integrated system to measure and regulate growth conditions, such as maintaining a desired cell culture density during an experiment. They have dedicated OD sensors as well as media replenishing and culture removal mechanisms to provide turbidostat, chemostat, or morbidostat modalities. These platforms usually have separate micro-controllers/processors to execute these functionalities either in a pre-programmed manner or a live run by receiving updated commands from a computer during the course of an experiment. **(D)** Photobioreactor illustration. This is a generic photo-bioreactor set-up where the cells are usually cultured in a glass (transparent) off-the-shelf bioreactor. Two or more high-power LED panels are placed around the bioreactor to provide sufficient illumination of the target cell culture as and when required. Some designs also involve LED strips wrapped around the bioreactor for better illumination.

One of the initial microscope-coupled projector-based optogenetic platforms [inspired by (Wang et al., 2007)] was employed for the spatio-temporal control of cell signalling by using a light-switchable protein-protein interaction by Levskaya et al., 2009. In their work, the authors engineered a light-controlled membrane recruitment module based on phytochrome-PIF interaction in mammalian cells. Using this module, they demonstrated spatially localized activation of Rho-family GTPases (Rac1, Cdc42, and RhoA) inducing cell protrusion at a desired cell-membrane location *via* a spatially localized delivery of light. The light-delivery platform they proposed used a digital micromirror device (DMD) projection set-up integrated with a fluorescence microscope. A similar set-up was also used by Toettcher et al., 2011c demonstrating feedback control over light-gated protein-protein (phytochrome and PIF-tagged inputs) interaction. They used live-cell measurements under the microscope to update light-illumination intensities delivered *via* a DMD-based device. DMDs are extensively used as spatial light modulators in commercial projection systems (Dudley et al., 2003), and here in these platforms DMDs facilitated projecting user-defined mask images onto the microscope sample plane where the target cells are present (Figure 3A). In the last decade, several improved microscope-coupled DMD or LCD projector setups have been designed and demonstrated in cellular optogenetic control applications (Zhu et al., 2012; Chait et al., 2017; Rullan et al., 2018; Kumar et al., 2021; Fox et al., 2022; Singh et al., 2021). In most of these platforms, a DMD (Rullan et al., 2018) or LCD (Chait et al., 2017) projector, along with additional optical elements like lenses, filters, *etc*., is placed at the episcopic illumination port of the microscope. The optical elements are chosen and positioned in such a way that the projection image is focused onto the microscope sample plane through the microscope objective. An illustration of a generic DMD based set-up is shown in Figure 3A. The intensity of a pixel in the input projection image determines the light intensity received by the region (in the microscope sample plane) where that particular pixel is being projected. This allows a user to target separate regions of the sample plane with different light intensities, thus enabling spatial light-stimulation intensity variation at intra-/inter-cellular resolution (within the microscope field of view). Together with suitable software packages integrated with cell segmentation, tracking and quantification routines (Rullan et al., 2018; Fox et al., 2022; Pedone et al., 2021), these platforms uniquely facilitate independent stimulation and real-time observation of multiple single cells (present in the microscope field of view) in parallel. In (Fox et al., 2022), the authors also discuss the effect of erosion in light-stimulation regions as DMD systems can induce illumination bleed-through on the microscope sample plane. Suitably-defined light-erosion around illumination regions helps in reducing the bleed-through, and improves the precision of single-cell targeting in dense cell regions in the microscope field of view.

All microscope-coupled optogenetic platforms (with/without projector) can, in principle, facilitate multiple light-stimulation wavelength operations. The DMD projector used in (Rullan et al., 2018) has red, green and blue LEDs as light sources allowing stimulation with possibly three separate wavelengths. Therefore, one can use these platforms when there are multiple orthogonal optogenetic constructs in the target cellular system. These platforms also require suitable cell culture set-ups compatible with imaging and light stimulation under the microscope. Projector-based platforms focus a 2-D projection image onto the microscope sample plane which requires the target cells to be present in a monolayer fashion under the microscope. Adherent mammalian cell lines, such as HEK-293, can be cultured in simple petri dishes (Toettcher et al., 2011c) or imaging-compatible multiwell plates for experiments with these platforms. For non-adherent cell types such as yeast cells, one needs to use culture set-ups in which cells are constrained to grow in a monolayer. In (Kumar et al., 2021), the authors cultured yeast cells in a monolayer for up to 6 h by sandwiching them between a solid nutrient agarose pad and a glass coverslip. Microfluidic chips provide another way for achieving constrained cell proliferation under the microscope as shown in several studies for yeast (Rullan et al., 2018; Durán et al., 2020), bacteria (Chait et al., 2017; Lugagne et al., 2017), and mammalian cells (Woodruff and Maerkl, 2016). These chips are suitable for long-term experiments as they facilitate constant replenishment of nutrients and removal of dead cells *via* medium flow. *In situ* time-varying media exchange or mixing is also possible with these chips (interested readers can refer to (Gale et al., 2018) for an in-depth discussion on microfluidics technology). One unique approach is culturing adherent cells directly on high-density arrays of microscopic organic LEDs (OLEDs) (Steude et al., 2016). This OLED set-up facilitates light induction at a very high spatial resolution and can be placed onto the microscope sample plane for imaging. Although innovative, this set-up is extremely delicate in the sense that one needs to have a very thin (of the order of a few micrometers) encapsulation layer over the OLEDs in order to protect the electronics from aqueous cell culture medium. There are also some emerging microscope-based technologies allowing spatial stimulation control in 3-D space. They are briefly introduced in “Other emerging techniques” section, later in this review.

As previously described, microscope-coupled optogenetic platforms provide light stimulation capability with a high spatial resolution (at the level of intra-/inter-cellular resolution), and also facilitate feedback control operation (by allowing both stimulation and fluorescence measurement) over the target cells. However, they have certain limitations that need to be addressed. First, it is difficult to controllably regulate cell density under a microscope. Cells continuously proliferate under favourable conditions, with the inevitable outcome that the microscope field of view will become fully confluent over time (dependent on cell doubling time), even if the experiment is started with only a few cells. This makes tracking, stimulating and imaging a single cell challenging in long-term optogenetic control experiments. One can use a microfluidic “mother machine” device (Chait et al., 2017; Durán et al., 2020) under the microscope to ensure single cell tracking in long-term experiments, but these special cell culturing devices are only compatible with bacterial or yeast cells. Additionally, while designing synthetic constructs in cells, one is restricted to using fluorescent proteins as a reporter system because observation on these optogenetic platforms is mostly carried out *via* fluorescence imaging. Furthermore, these optogenetic platforms come with a hefty financial burden. Advanced microscopes, which can cost more than $100k, are one of the most expensive equipments in a biology lab, and the commercially available pattern projector set-ups (for example, Mosaic - Andor Oxford Instruments, Polygon - Mightex, DMD module - Nikon, *etc*.), capable of coupling with a microscope, are also expensive (∼ *$*25–60k).

### 2.2 LED-Array Light Induction Device

For applications not requiring light stimulation at single-cell resolution, there are also custom-designed LED arrays or matrices adapted for population-level induction of cells grown in microwell plates. One simple design was used by Ye et al., 2011 where the authors demonstrated light-inducible transgene expression in mammalian cells. Their device comprises a matrix of 6 or 12 blue LEDs (placed above 6- or 12-well cell culture plates) connected with a regulated DC power supply controlled by Labview software from a computer. A detailed protocol for the construction of an improved programmable device (intended for 12-well format plates) has been provided by Tucker et al., 2014. This design provides independent programming of upto three LED outputs. Additionally, it includes LCD display interface and keypad for setting the duration, interval and intensity of LED pulses without needing any computer for configuration. Although innovative, these simple devices do not provide optical isolation between adjacent well LEDs, and hence do not offer independent induction control among multiple wells. Researchers have proposed a multitude of other evolved and refined optogenetic platforms for multiwell plates. A general architecture of these platforms is illustrated in Figure 3B.

In (Mertiri et al., 2011; Chen et al., 2012) inspired by (Johnson and Sheldon 2007), the authors presented an optical microplate system having an 8 × 12 matrix of 96 LEDs (128 brightness levels) suitable for light induction in a 96-well plate from below, and demonstrated its use in the high-throughput screening of algal photosynthesis. By using a standard black 96-well plate to encase the LED matrix, this design ensures very little spillover between adjacent LEDs, and hence each cell culture-containing well of a 96-well plate (black walls and transparent bottom), placed on top of the device receives light from separate LEDs underneath. These LEDs are individually controlled (time and intensity) *via* a serial microcontroller, thus facilitating parallel runs of high-throughput experiments. A graphical user interface (GUI) in Labview software is used to program different light patterns on the device. This optical microplate design was further augmented by Davidson et al., 2013 with multi-color LEDs providing two-color control in each well. Further, Hennemann et al., 2018 proposed a set-up where three fixed-color channel LEDs (470, 525, and 620 nm) are used in an 8 × 8 layout (for 64 separate wells) that can achieve 256 brightness levels. They also designed a python-based GUI for easy configuration of LEDs for light induction experiments. An improved and refined design in optoPlate-96 (Bugaj and Lim, 2019) facilitates one- to three-color illumination (4,096 brightness levels) for individual wells of a 96-well plate (or neighbouring groups of four wells in a 384-well plate). As per user requirements, different wavelength LEDs can be substituted on this modular platform. Additionally, a dedicated active heat management system comprising a heat sink and a cooling fan has been incorporated in this design to minimize heat generation from on-board electronics.

Similar optogenetic platforms have been proposed and demonstrated in applications for cell/tissue cultures in 24-well plates. In (Müller et al., 2014), the authors used a simple illumination box having LEDs mounted on the inner lid of an enclosed box in which the cell/tissue culture well plate can be placed for stimulation. Here, the wells are not optically separated, and thus cannot be independently illuminated. Platforms devised in (Hannanta-Anan and Chow, 2016; Lee et al., 2013; Gerhardt et al., 2016) have improved designs with adequate optical separation through black walls and independent control over individual well LEDs in the 24-well black-wall transparent-bottom plate. In the device proposed by Hannanta-Anan and Chow (2016), not individual LEDs but columns of four LEDs (with six columns in total) are independently controllable. The Light Plate Apparatus (LPA) proposed by Gerhardt et al., 2016 has two configurable and independent LEDs for each well of the 24-well plate, and each LED intensity can be adjusted with 4,096 brightness levels. This device uniquely uses solder-free sockets for LEDs which makes it much easier to change/replace LEDs without any wear and tear. Gerhardt et al., 2016 also designed a user-friendly web tool to program the device for static (constant light stimulation) as well as dynamic (periodically varying light stimulation) experiments.

An improved design (LAVA), intended for independent well stimulation of 24-well or 96-well culture plates, has been recently proposed by Repina et al., 2020. Here, the authors have shown a detailed characterization and quantification of illumination uniformity, device overheating, phototoxicity, and spatio-temporal light-patterning resolutions in their proposed design. Provisions for improved operation concerning these modalities have also been incorporated in the set-up. The authors also demonstrated spatial patterning (with ∼ 100 *μ*m resolution) using a photomask on this platform. This optogenetic device can be configured for an experiment *via* an easy-to-use and intuitive GUI, which allows users to set time-varying and complex light intensity patterns for each independent well.

These multiwell plate optogenetic platforms usually consist of low-cost widely-available LEDs which always have brightness differences due to variation in the manufacturing process. This implies that two LEDs of the same type will always display slightly different light intensities, even when powered by the same electrical current. This intensity difference may lead to discrepancies when replicating light stimulation experiments. That is why the LEDs in these set-ups have to be characterized and power-corrected to reduce intensity variation between them (and in turn reduce the well to well variation). Mertiri et al., 2011 and Chen et al., 2012 followed a manual calibration procedure by manually measuring with a light meter the light intensity each well receives, and scaling the power setting in each well by a suitable calibration factor. In (Grødem et al., 2020), the authors propose an automated calibration method for optoPlate (Bugaj and Lim, 2019) LEDs. They use a programmable microscope stage and optical power meter to automatically measure all LEDs on the device and then compute the required calibration values needed to adjust the electrical current supplied to each LED in order to reduce the light intensity variation between LEDs. In (Gerhardt et al., 2016), the authors describe two calibration methods for their LPA device: image analysis (for calibrating LEDs of same wavelength) and probe spectrometer (for calibrating LEDs of different wavelength) methods. The first method involves capturing top-down images of the device with LEDs lit at a constant intensity, and then further analysing the pixel intensities in the image using a MATLAB script to measure differences in light intensities between wells. These pixel intensity values are then used to compute grayscale adjustment and dot correction parameters for each LED. The other probe spectrometer method involves measuring the photon flux of each LED using a probe spectrometer aligned *via* a 3D printed probe adapter, and then adjusting dot correction based on measured values. These calibration procedures have been shown to reduce LED brightness variability to <1% . A unique provision for calibrating time-steps is also available in the LPA design.

In contrast to microscope-coupled platforms, these LED-array devices are low-cost (under *$*1k), and can be designed in-house with readily-available components. Building and assembling these set-ups require only basic knowledge and experience with electronics and mechanical design tools. There are also some off-the-shelf commercially available options, such as Lumos from Axion Biosystems, but they are expensive (∼ *$*13–26k for Lumos) compared to previously discussed custom-designed devices. The small form-factor of these platforms makes them easy to transport; and they can also be placed inside incubators, thus ensuring favourable conditions for cell cultures during the experiment. Some of these set-ups also incorporate a thermal management system (heat sink, cooling fan, *etc*.), for example in (Bugaj and Lim, 2019; Repina et al., 2020), to negate the effect of heat generated by LEDs and electronic circuitry onto the target cells under stimulation. In most of these devices, the illuminating LEDs can be substituted with the wavelengths required for individual application, and some of the improved ones (mentioned previously) also facilitate up to three wavelength inductions in the same experiment. These set-ups can also be fit with diffuser films/sheets, such as in (Gerhardt et al., 2016), to ensure homogeneous illumination inside the well. They are also high throughput devices, and depending on the number of wells in the multiwell plate being used, one can perform that many parallel studies with cell cultures. As mentioned previously, these platforms (unlike microscope-coupled platforms) do not provide precise spatial control over light delivery. But, one can use simple photomasks to achieve a desired spatial-pattern light illumination onto lawns of cells cultured in well plates or petri dishes (Levskaya et al., 2005; Beyer et al., 2015; Romano et al., 2021; Morales et al., 2021). Using illumination through photomasks, the authors in (Levskaya et al., 2005; Romano et al., 2021) have also demonstrated creating a high-definition high-contrast chemical image (bacteriograph) of the projected light mask pattern similar to images captured on camera films.

One major disadvantage of these LED-array platforms is their inability to perform measurements during an experiment. One can only obtain a steady-state final measurement after light induction is finished; one cannot perform dynamic or periodic time-point measurements. Hence, these devices do not, in general, offer feedback control capability. However, Chen et al., 2020 use the optoPlate design (Bugaj and Lim, 2019) to achieve simultaneous light-stimulation of every cell culture in a 96-well matriplate while intermittently imaging under a widefield microscope. One can place these optoPlates on top of the 96-well sample plate to achieve illumination from above and imaging from below when placed under an inverted microscope. Recently, researchers in (Soffer et al., 2021) have proposed an innovative light delivery system (RT-OGENE) to allow both microwell plate illumination with a 2 × 2 RGB LED matrix (so only four wells can be illuminated simultaneously) and fluorescence/absorbance measurement with a simple camera without needing an expensive microscopy platform. The set-up involves a camera and a long-pass filter placed on top of the cell culture well-plate with optical filters and illumination LEDs placed underneath. This whole platform is placed inside a custom-designed incubation chamber fit with temperature control system. By having both stimulation and measurement devices available on this platform, they demonstrated closed-loop feedback control of a CcaR-CcaS optogenetic construct in *Escherichia coli*. In addition to multiwell plates, this device is also shown to be compatible with previously described microfluidic cell culture platforms. Another interesting approach of augmenting a multimode microplate reader with illumination sources has been explored in (Richter et al., 2015). Here, the authors attached a fiber-optics waveguide with the injection cylinder of Tecan Infinite M200 pro multimode microplate reader. By default, in this plate reader the injection cylinder can inject liquids into defined wells of the microwell sample plate; but with this proposed modification one can also illuminate those defined wells *via* a waveguide. A separate microcontroller was integrated with the plate reader control software to define illumination position and wavelength (the waveguide was connected to an input LED light source containing eight different LEDs) in the set-up. Although this platform has the potential to facilitate feedback control operation (stimulation *via* waveguide and measurement *via* plate reader) over multiwell sample plates, the wells can only be stimulated sequentially, thus limiting its throughput. At the same time, Tecan microplate readers are very expensive (∼ *$*40k), thus limiting its adoption.

## 3 Medium-Volume Culture Platforms

This section spans optogenetic platforms with intended target cell culture volumes of a few to hundreds of milliliters. These volumes are usually considered in studies where certain constraints on growth conditions or cell density are needed during long-term experiments. For example, in the optogenetic experiments in (Aditya et al., 2021), cells needed to be continuously maintained in exponential growth phase. Low-volume culture platforms are not suitable for those studies as it is difficult to regulate cell density at such low volumes. Most of the platforms described here are coupled with turbidostat, chemostat, or morbidostat set-ups in order to facilitate operation in those conditions. A generic architecture of these platforms is shown in Figure 3C.

One of the initial designs in this category was proposed by Milias-Argeitis et al., 2011 in their pioneering work of demonstrating *in silico* feedback control of gene expression in yeast. This set-up involved a custom-built light delivery system with a light pulser device (having two triplets, red and far-red wavelengths, of high power LEDs) and an electronics box containing LED driver electronics which can be connected with a computer *via* a multifunction DAQ (Data Acquisition) board. The custom-designed electronics provided both manual as well as computer-controlled (needed for feedback control experiments) operation of the device. This system can be used both with multiwell cell culture plates (without independent illumination of separate wells) by placing sample plates underneath the light pulser device (Milias-Argeitis et al., 2011), and with glass/transparent tube cultures (Ruess et al., 2015). The authors in (Ruess et al., 2015) used this system with a hot plate magnetic stirrer to maintain a desired cell culture temperature and provide sufficient aeration to the target culture. They also diluted the culture manually at regular intervals to keep the cell density within reasonable bounds during an experiment. Another simple design with a higher-throughput set-up (capable of running 15 tubes independently in parallel) was used by Baumschlager et al., 2017 (also in (Benzinger and Khammash, 2018)). This set-up comprises a water bath (to maintain a desired temperature) having custom-built 15-tube holders, which can hold 25-ml glass cell culture tubes, each fitted with a magnetic stirring device and a custom-made LED pad (with a white diffusion filter to ensure homogeneous illumination) located underneath each culture tube. This set-up allows for having a pre-programmed illumination sequence for each tube in an experiment, but does not allow for the real-time change of LED intensity during the experiment, and thus doesn’t have feedback control operation capability. A similar but improved Light Tube Array (LTA) platform was proposed by Olson et al., 2014 which is capable of providing an isolated optical environment for 64 standard 14-ml culture tubes. Each tube can be illuminated with four different LEDs independently with the given LED driver electronics that can be pre-programmed with light-intensity sequences. Although the set-up does not include any stirring or temperature control mechanism in itself, the whole tube rack can be placed in a suitably-sized shaking incubator.

Several other innovative and advanced platforms combining multiple functionalities and automation principles have been proposed as well as demonstrated in various applications, notably feedback control over cellular processes (owing to the availability of both light-stimulation and measurement devices on these platforms). In (Melendez et al., 2014) and improved in (Stewart and McClean, 2017), the authors exhibited controlling intracellular protein concentration with a CRY2-CIB1 optogenetic construct in yeast by using their automated culturing optogenetic platform. The set-up they proposed involves a 27-ml working volume transparent culture vessel where cells can either be grown in batch culture or maintained as continuous culture for several days in chemostat mode by using the automated media influx and culture efflux system. Provisions for temperature control, agitation and aeration to oxygenate cells in the vessel are available on this platform. A separate microscope-based measurement apparatus is also integrated with the set-up, allowing fluorescence imaging and quantification of single cells in the culture by running them through a single channel microfluidic flow cell mounted on the microscope. Light induction in the culture vessel is performed with a high-powered blue LED, all controlled by a computer *via* a custom-designed control board. Another automated optogenetic platform demonstrated by Milias-Argeitis et al., 2016 employs a custom-designed turbidostat to maintain cell culture at a desired optical density (OD), and an automated sampler to facilitate real-time measurement *via* flow cytometer. This platform also has integrated culture incubation, LED-based light induction, and a culture stirring system for proper aeration in the 25-ml glass centrifuge cell-culture tube. By using computer control over the whole apparatus, Milias-Argeitis et al., 2016 exhibited optogenetic cell growth-rate control of a culture of bacterial cells for over 24 h. Another similar platform was proposed by Harrigan et al., 2018 which comprises eight (in two incubator arrays of four each) or more (expandable) separate temperature-controlled and magnetically-stirred chambers for 50-ml conical cell culture tubes and LED stimulation. The authors also designed an automated integrated liquid handling module capable of moving cell culture from individual chambers to a flow cytometer for measurement or to a waste reservoir for dilution as well as replenishing the individual chambers with fresh media.

Recently, researchers have put forth various innovative and compact optogenetic platform designs pertaining to this medium-volume culture category. One particularly unique design, Chi.Bio, was presented by Steel et al., 2020 which establishes a very compact and low-cost approach of optogenetic stimulation and *in situ* fluorescence measurements. Their system is divided into reactors, pump boards and mini control computers. The reactor is a compact housing for 30-ml clear glass test tubes (open to the atmosphere), and it has integrated tunable light-induction LEDs, temperature control and stirring systems, an OD sensor, and a spectrometer for measuring multiple fluorescence proteins *in situ*. One pump board per reactor is needed to achieve media or culture flow to/from the reactor culture tube providing turbidostat/chemostat/morbidostat operation capability. Furthermore, the mini control computer can control eight such integrated reactor pump board set-ups independently in parallel. The software stack enables users to control and monitor any peripherals, and set-up experiments *via* a user-friendly web user interface. Any advanced procedures and computations in an automated experiment, for example feedback control algorithms, are easily implementable *via* their python framework. One downside of this platform is the low sensitivity of its low-cost integrated chip-based spectrometer compared to much more expensive flow-cytometers. This might be a limiting factor in some applications with synthetic genetic constructs requiring very sensitive fluorescence measurements. But one can also integrate separate liquid handling modules with this set-up to facilitate flow-cytometric or microscopic measurements in addition to the *in situ* fluorescence measurement. The ReacSight strategy proposed by Bertaux et al., 2021a offers such an enhancement. This is a generic and flexible strategy for adding different instruments in the automated measurement framework of different optogenetic platforms. Bertaux et al., 2021a also demonstrated this strategy with Chi.Bio reactors as an example, and this combined platform of ReacSight and Chi.Bio has been further used in some recent optogenetic control studies (Aditya et al., 2021, 2022).

Another interesting optogenetic platform with a custom-designed multicolor fluorescence monitoring device was proposed by Pen et al., 2021. Here, the cell culture chamber is a continuous cell culturing module (allowing turbidostat or chemostat mode operation) adapted for a 30-ml cylindrical glass vial with a gasket cap (for gas handling functions), and includes temperature control and magnetic stirring systems. Additionally, this set-up by Pen et al., 2021 also has a separate module for *in situ* fluorescence measurement. There is a provision of dipping a multifiber (optic bundle) probe into the cell culture through the lid which allows excitation light propagation from excitation LEDs onto the cell culture and subsequent collection of fluorescence emission signals which are then guided towards a microPMT (Photomultiplier tubes) for fluorescence measurements. These PMTs are expensive but have a higher sensitivity and higher dynamic range compared to the fluorescence sensor used in Chi.Bio. This platform can also be modified and used for light stimulation of the cell culture *via* the fluorescence excitation LEDs.

Similar to the LED-array light induction devices discussed in the previous section, these optogentic platforms are only intended for target stimulation and observation on a population level, meaning they cannot be employed for single-cell studies. Standalone optogenetic platforms without a measurement device can cost around *$*10k or less for 10–16 parallel throughput set-ups. However, to achieve feedback control, one would need to integrate them with other measurement devices like flow-cytometer or microscope which are very expensive. As mentioned previously, the Chi.Bio set-up (Steel et al., 2020) does offer a low-cost integrated spectrometer, but it is not suitable for measuring weak fluorescence signals. Unlike low-volume culture platforms, most of these devices have integrated turbidostat, chemostat, or morbidostat operation set-ups which can be used to maintain growth conditions and regulate cell densities in the target culture during an optogenetic experiment. Moreover, most of these platforms can only work with suspension cell cultures. In the non-optogenetic biology literature, there has been extensive development of turbidostat/chemostat/morbidostat set-ups; therefore, a wide variety of designs, intended for different applications, are now available (Toprak et al., 2013; Miller et al., 2013; Takahashi et al., 2015; Hoffmann et al., 2017; Wong et al., 2018; Bergenholm et al., 2019; McGeachy et al., 2019; Gopalakrishnan et al., 2020). With some basic electronics know-how, one can easily integrate light-stimulation LEDs or other light sources into these already existing devices to achieve optogenetic stimulation.

## 4 Large-Volume Culture Platforms

Here we consider optogenetic platforms aimed at suspension cell culture volumes beyond hundreds of milliliters. These large volumes are usually handled in large-scale bioreactors which are one of the staple devices in biochemical/bioprocess industries (Najafpour, 2015). Given the scope of this review article, we will restrict our discussion to bioreactor platforms employed in academic research.

There have been a number of incremental studies on metabolic engineering and protein production regulation in lab-scale bioreactors. One of the major goals of metabolic engineering research today is to maximize the yield of a given bioproduction process. To achieve this goal, there is an ever-growing interest in the synthetic biology community to build genetic constructs allowing for dynamic control of metabolism (Venayak et al., 2015; Lynch, 2016; Ni et al., 2021). The idea is to introduce externally inducible systems in metabolic pathways with different induction strategies (Lalwani et al., 2018; Bertaux et al., 2021b). Several induction strategies (e.g. chemical induction, temperature control *etc*.) have been explored in this context. A list of these strategies, pertaining to *Escherichia coli* and *Saccharomyces cerevisiae* cell-cultures, has been summarized in (Lalwani et al., 2018). With the recent development in optogenetics and related genetic tools, one can not help but foresee its application in metabolic engineering with its unique benefits (light induction is inexpensive and provides precise temporal control) (Carrasco-López et al., 2020; Pouzet et al., 2020). However, this strategy of using light induction for efficient bioproduction is still in its nascent phase of realization, and only a few studies with simple light delivery devices have been carried out in recent years.

In (Zhao et al., 2018), the authors demonstrated production of isobutanol and methyl-1-butanol *via* light stimulation of an engineered *S. cerevisiae* culture. In their study, they used 500-ml, 2-L and 5-L capacity bioreactors with custom-designed light delivery set-ups. Multiple (2 for 500-ml and three for 2-L) LED matrix panels were used to surround the first two (500-ml and 2-L) transparent glass bioreactors, while multiple blue LED strips were wrapped around the 5-L bioreactor to achieve light stimulation of respective cell-cultures. A similar set-up for a 2-L bioreactor was used by Lalwani et al., 2021 to demonstrate light-induced mevalonate and isobutanol production in engineered *E. coli*. In (Zhao et al., 2021), the authors presented a series of sensitive optogenetic circuits (OptoAMP) and demonstrated their ability to enhance the production of lactic acid, isobutanol, and naringenin on a photo-bioreactor platform. They used a commercial high power LED-matrix panel to illuminate the 5-L bioreactor in their experiments. These simple light delivery devices are inexpensive and can be coupled with other suitable (transparent) bioreactors which are usually expensive on their own. A representative design of these platforms is illustrated in Figure 3D.

One problem encountered when illuminating a large volume culture is insufficient penetration of light at high cell densities (Carrasco-López et al., 2020). This challenge can be overcome by either improving the sensitivity of optogenetic circuits (Zhao et al., 2021), risking a reduction in dynamic range and an increase in leaky dark activation, or by improving the light delivery system. One can design the inside of a bioreactor with provisions for additional light sources to enhance light penetration, for example, having multiple LED rods dipped in the target cell culture *via* suitably designed lids. Similar design strategies have been proposed by Carrasco-López et al., 2020 in their opinion article, but these designs have not been implemented yet. Within the realm of microalgae biotechnology, large photobioreactors are commonly used for microalgae cultivation. One can also employ their designs (Posten, 2009; Chen et al., 2011) for bioreactor illumination with little or no modifications. Furthermore, to provide feedback control, a liquid handling module similar to the design proposed by Harrigan et al., 2018 can be used to move samples from the bioreactor to a suitable measurement device such as flow-cytometer. To the best of our knowledge, no optogenetic feedback control as envisioned in (Carrasco-López et al., 2020; Pouzet et al., 2020) has been demonstrated yet with these bioreactor platforms.

## 5 Other Emerging Techniques

Apart from the devices and systems covered in the previously discussed three categories (low-, medium-, and high-volume culture platforms), there are some other innovative and unique designs which were developed for specific applications. We would like to mention a few of these platforms which may have potential utility in emerging optogenetic applications.

A compact light delivery device (LED Thermo Flow) that can be directly connected to flow-cytometer injection ports was described by Brenker et al., 2016. In this design, a glass FACS tube containing target cell culture is inserted into a custom-built cylindrical sleeve. The sleeve contains multiple wavelength LEDs with manually-controllable intensities surrounded by water which is constantly circulated *via* a heated water pump to maintain the sample temperature at a desired value. The FACS tube, together with the sleeve, is then connected to a flow-cytometer injection port for direct measurement. This allows one to measure real-time effects of optogenetic stimulation and deduce kinetics of optogenetic tools.

In all the optogenetic platforms described previously, light illumination was targeted for cells growing in any cell culture lab-platform but not for cells proliferating within a live animal. In (Ye et al., 2011), the authors devised a system to provide light-stimulation to mammalian cells (engineered with a desired optogenetic construct) implanted intraperitoneally in mice. In their set-up, light from a high-brightness LED is delivered to the cells in hollow-fibre implants *via* a multimode silica glass optical fibre. The LED is powered by a regulated DC power supply whose output voltage (in turn, the LED light intensity) can be modulated *via* Labview software running on a computer.

All the platforms discussed so far in this review facilitated either precise spatial 2-D illumination (microscope-coupled platforms) or full 3-D homogeneous illumination with no spatial control. One of the emerging light induction techniques involves stimulating samples in 3-D with spatial precision. Holography-inspired multiphoton photo-excitation (3D-SHOT) (Pégard et al., 2017), DMD-based patterned illumination in lightsheet microscopy (Jiao et al., 2018), and three-dimensional multi-site random access photostimulation (3D-MAP) (Xue et al., 2022) are some of the recently proposed designs in this direction. These platforms have the potential to aid artificial tissue engineering or organoid development *via* optogenetic induction. To the best of our knowledge, these new techniques have only been applied in neuroscience research such as whole-brain neuronal activity studies in larval zebrafish (Jiao et al., 2018). Their application with non-neuronal cells or tissues is yet to be explored.

## 6 *In Silico* Feedback Control Strategies

Feedback control is a key concept in the control theory literature. It has a wide range of applications in our day to day life, from a thermostat controlling room temperature to the autopilot maintaining airplane attitude. Feedback control over a process requires a mechanism to tweak the process as well as a pathway to measure the process behaviour. Here, we will briefly discuss different feedback control methodologies pertaining to cellular biology contexts where the tweaking/stimulation of a target process (such as gene expression) is enabled by optogenetics. A brief introduction and description of optogenetic *in silico* feedback control procedure has been laid out previously in the introduction section, and a related illustration is shown in Figure 2.

**FIGURE 4:**
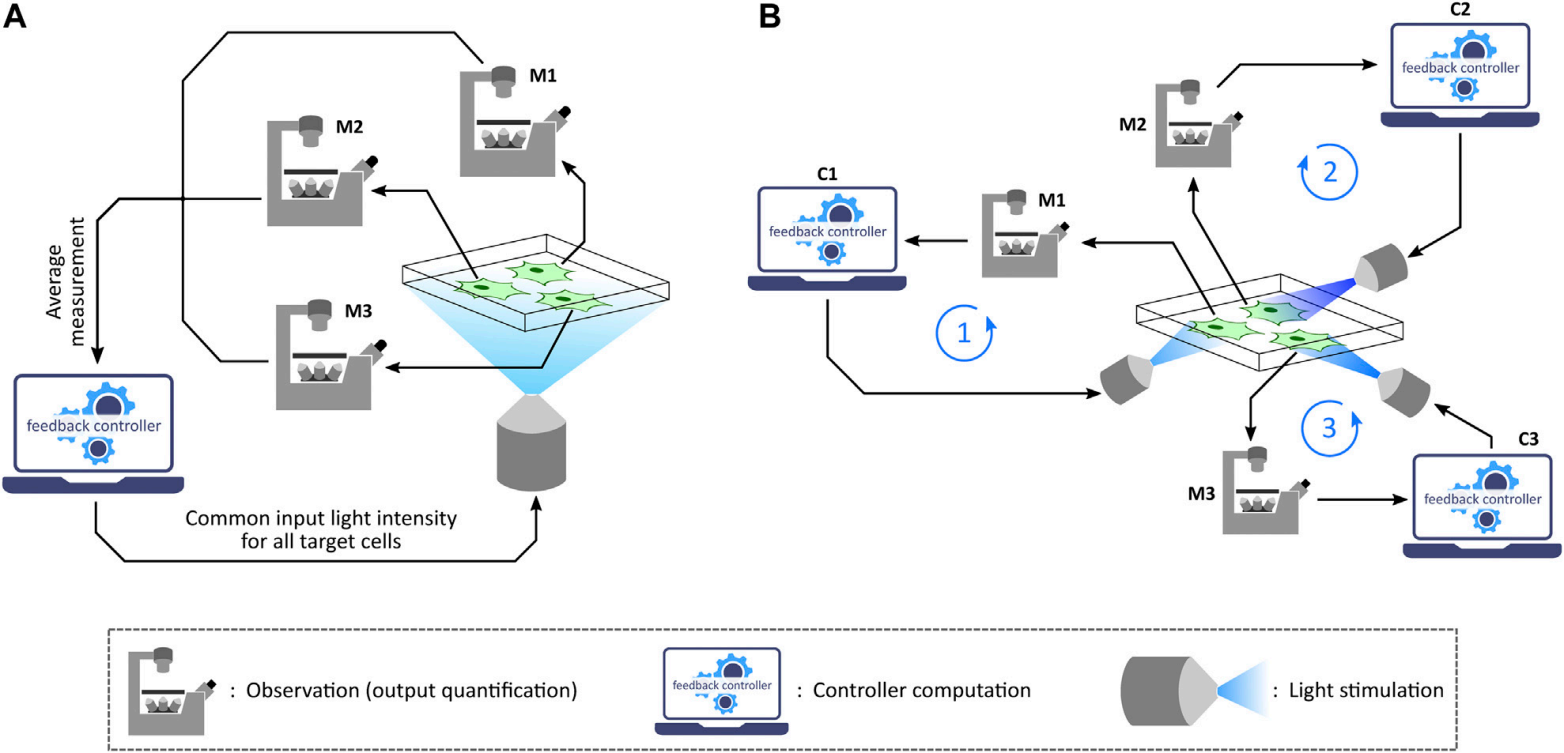
Population control and single-cell control. **(A)** In the population feedback control strategy, a statistically average measurement from all the target cells in a culture is sent to a computer. The feedback controller is then simulated over the average output value and then a common input light intensity is computed. This intensity is applied to all the target cells in the culture *via* a suitable illumination set-up. Note: M1, M2, and M3 represent the same microscope. **(B)** In the single-cell feedback control strategy, each cell undergoes independent and parallel treatment having its own feedback loop in action. Once a fluorescence image is captured under the microscope, the image is segmented to quantify output measurement from single cells. These quantified outputs are then sent to separate feedback controllers corresponding to individual cells. Input light intensities for each cell are computed and also applied to the corresponding cells separately *via* a projector-based set-up integrated with the microscope. Note: M1, M2, and M3 represent the same microscope; C1, C2, and C3 represent the same control computer.

Depending on the application as well as the capability of the optogenetic platform used, one can implement *in silico* feedback control over a target cell culture in two different settings: population control (Milias-Argeitis et al., 2011; Melendez et al., 2014; Milias-Argeitis et al., 2016; Harrigan et al., 2018) and single-cell control (Rullan et al., 2018; Kumar et al., 2021; Fox et al., 2022), as illustrated in Figure 4. Population control involves an aggregate measurement of the target cell culture and stimulation with a common input light intensity for all the cells in every measurement-computation-stimulation cycle. This can be achieved with those optogenetic platforms which are integrated with a measurement device (assuming that the required controller computation over measurements to compute the input light intensity can be performed on a computer). Meanwhile, exhibiting a reduced cell-to-cell output variability compared to population control (Rullan et al., 2018), single-cell control involves measuring single-cell outputs in a culture, running controller computations for each cell based on its quantified output, and then applying the computed light intensities to the respective target cells independently. This setting can be employed with microscope-coupled projector-based optogenetic platforms discussed previously.

Another critical aspect of *in silico* feedback control application is the type of controller algorithms employed in the computation. This controller algorithm has the task of computing an input light-intensity (which the target cell culture should be stimulated with) based on output measurements obtained from the target cell culture (in every measurement-computation-stimulation cycle), in order to achieve a desired output behaviour (Figure 2), for example, driving nascent RNA count to a desired set-point value (Kumar et al., 2021). One of the initial demonstrations of optogenetic *in silico* feedback control was realized by Milias-Argeitis et al., 2011. With their custom-designed optogenetic platform, the authors demonstrated robust regulation (desired set-point tracking) of gene expression fold change in genetically engineered *S. cerevisiae* by using an *in silico* feedback controller implementing Model Predictive Control (MPC) strategy. Further, Milias-Argeitis et al., 2016 carried out optogenetic *in silico* feedback regulation of gene expression as well as cell growth in suitably engineered *E. coli* cultures. They implemented two different control algorithms: Proportional-Integral (PI) and MPC. PI controllers have a very simple implementation offering a control input (light-intensity) value which is proportional to the sum of *1*) the error between a desired set-point and the current measured output, and *2*) the time-integral of this error. These PI controllers guarantee zero error at steady-state when the set-point is constant over time, but they are not suitable for tracking time-varying set-points. On the other hand, MPCs are capable of tracking dynamic set-points but they require a model of the target process in their implementation. Further technical details and a comparison between these two controllers are described in (Milias-Argeitis et al., 2016). Another approach using a Proportional-Integral-Derivative (PID) controller has been proposed by Soffer et al., 2021 where the authors employed a model-based design for tuning controller parameters. Other *in silico* controller implementations such as PI control (Toettcher et al., 2011c), bang-bang control (Melendez et al., 2014), MPC (Harrigan et al., 2018), and integral feedback (Rullan et al., 2018) have been demonstrated with their respective optogenetic platforms and target processes. Interested readers can refer to (Grosenick et al., 2015) for a list and brief descriptions of different controller types which can be realized in *in silico* control architecture for controlling any suitable target cellular process. We would like to note here that this list is formulated in the context of neuroscience applications, but similar conclusions can be extended to non-neuronal cellular applications as well. A similar list of feedback controllers (including their advantages and disadvantages) along with some examples in non-neuronal studies can be found in (Carrasco-López et al., 2020).

**FIGURE 5:**
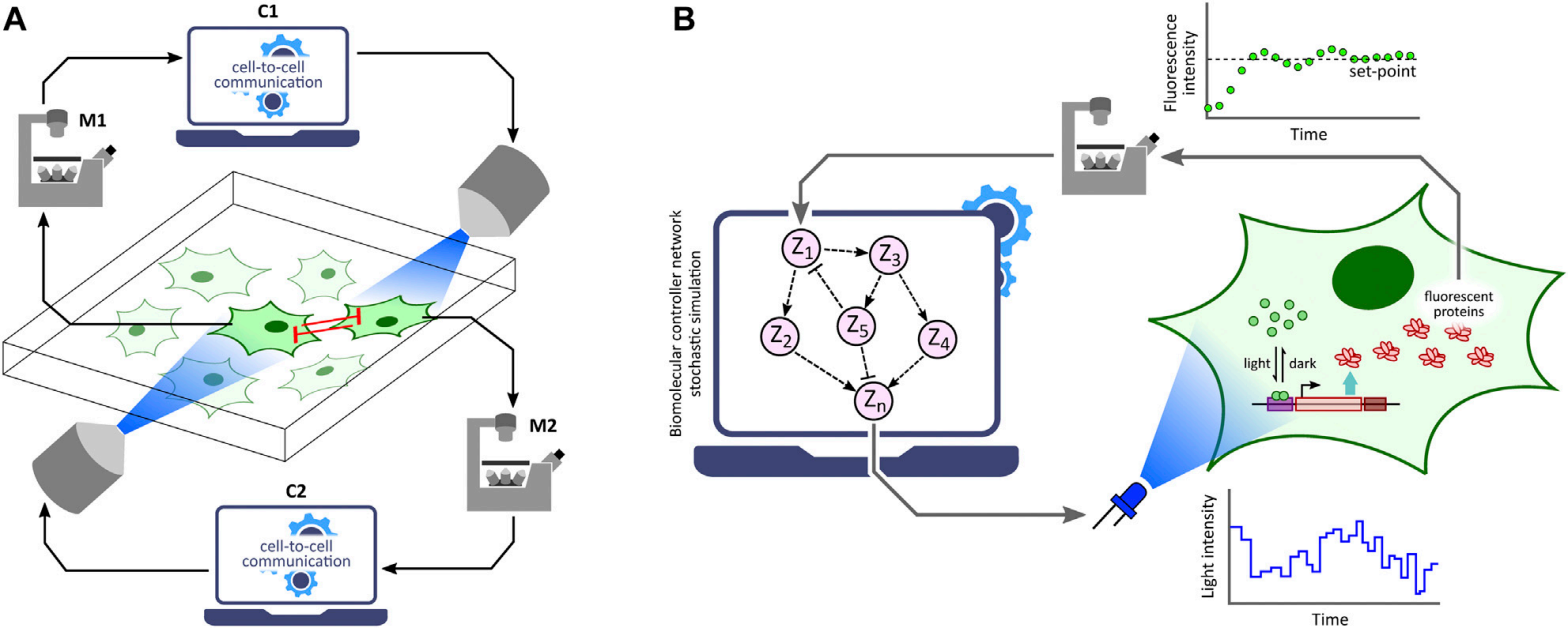
Applications of optogenetic *in silico* feedback control. **(A)** Cell-to-cell communication studies. Cellular outputs from neighboring cells are used to determine the light stimulation intensity of a target cell. Using this framework, lateral inhibition between neighboring cells has been shown to produce emerging checkerboard patterns in a 2-D grid of cells (Perkins et al., 2020). Note: M1 and M2 represent the same microscope; C1 and C2 represent the same control computer. **(B)** Rapid prototyping of biomolecular controller motifs. Stochastic simulation of biomolecular controller networks is run in the *in silico* feedback control framework with an *in vivo* target network. This allows rapid benchmarking and characterization of these controller motifs without requiring their *in vivo* implementations (Kumar et al., 2021).

Optogenetic *in silico* feedback control has numerous emerging applications in biotechnology, biomedicine, bioproduction and various synthetic biology studies (Chen et al., 2013; Liu et al., 2018; Lugagne and Dunlop, 2019; Hartmann et al., 2020; Banderas et al., 2020; Carrasco-López et al., 2020; Pouzet et al., 2020; Perrino et al., 2021; Ruolo et al., 2021). Formation of emerging patterns from lateral inhibition *via* an optogenetic *in silico* feedback approach to emulate cell-to-cell communication (Figure 5A) was demonstrated by Perkins et al., 2020 using the optogenetic platform described in Rullan et al., 2018. Furthermore, Kumar et al., 2021 implemented several *in silico* biomolecular controller motifs in stochastic setting and exhibited transcription regulation in suitably engineered *S. cerevisiae via* single-cell optogenetics control. With this *in vivo* (transcription in yeast) - *in silico* (stochastic controller) hybrid closed-loop set-up, called *Cyberloop* (Figure 5B), they showed characterization and rapid-prototyping of autocatalytic integral (Briat et al., 2016a), antithetic integral (Briat et al., 2016b), antithetic integral rein (Gupta and Khammash, 2019), and biomolecular PID (Filo et al., 2022) controller motifs. These characterization results provide useful insights for optimal controller performance, which can further guide *in vivo* implementations of these biomolecular controller motifs.

## 7 Conclusion

With the advent and ongoing development of various synthetic optogenetic tools that can be implemented in various cell types, a number of hardware-software platform designs have been co-developed. As discussed in this review, these optogenetic platforms have varying features and design methodologies depending on the application, target cell type, and target cell culture volumes. Their complexities range from simple LED strips to full-fledged microscope-coupled 3D hologram generators. Various simple set-ups, mentioned in this review, comprise readily available low-cost components, and can be easily built/assembled in basic research labs equipped with elementary electronic tools and minimal mechanical design expertise. They have the potential to reduce barriers for carrying out advanced optogenetics research with minimal resources. When integrated with an appropriate measurement device, some of these platforms also provide optogenetic *in silico* feedback control (Cybergenetics) capability, with which different feedback control strategies can be implemented and investigated. This capability has a wide scope of applications in biotechnology and synthetic biology studies.
